# OhrR of *Mycobacterium smegmatis* senses and responds to intracellular organic hydroperoxide stress

**DOI:** 10.1038/s41598-017-03819-1

**Published:** 2017-06-20

**Authors:** Omar A. Garnica, Kishore Das, Subramanian Dhandayuthapani

**Affiliations:** Center of Emphasis in Infectious Diseases and Department of Biomedical Sciences, Paul L. Foster School of Medicine, Texas Tech University Health Sciences Center El Paso, Texas, 79905 USA

## Abstract

Organic hydroperoxide reductase regulator (OhrR) in bacteria is a sensor for organic hydroperoxide stress and a transcriptional regulator for the enzyme organic hydroperoxide reductase (Ohr). In this study we investigated, using a GFP reporter system, whether *Mycobacterium smegmatis* OhrR has the ability to sense and respond to intracellular organic hydroperoxide stress. It was observed that *M*. *smegmatis* strains bearing the *pohr*-*gfpuv* fusion construct were able to express GFP only in the absence of an intact *ohrR* gene, but not in its presence. However, GFP expression in the strain bearing *pohr*-*gfpuv* with an intact *ohrR* gene could be induced by organic hydroperoxides *in vitro* and in the intracellular environment upon ingestion of the bacteria by macrophages; indicating that OhrR responds not only to *in vitro* but also to intracellular organic hydroperoxide stress. Further, the intracellular expression of *pohr* driven GFP in this strain could be abolished by replacing the intact *ohrR* gene with a mutant *ohrR* gene modified for N-terminal Cysteine (Cys) residue, suggesting that OhrR senses intracellular organic hydroperoxides through Cys residue. This is the first report demonstrating the ability of OhrR to sense intracellular organic hydroperoxides.

## Introduction

Although all living cells generate superoxide anions (O_2_^−^) inadvertently when oxygen molecules (O_2_) collide with redox enzymes containing flavins^1^, the phagocytes of the innate immune system such as macrophages and neutrophils, which engulf invading microbes and destroy them, have a dedicated enzyme to produce superoxide^2, 3^. This enzyme NADPH-oxidase, also known as phagocyte oxidase (Phox) or NOX2, has five subunits that are assembled on the membrane of the phagosomes during their maturation into phagolysosomes. NADPH oxidase transfers electrons from NADPH to O_2_ across the phagosomal membrane to generate O_2_^−^ anions inside the phagosomal compartment^4^. Consequently, this O_2_^−^ serves as the basis for the generation of several other reactive oxygen species (ROS) within the phagosomes. First, the O_2_^−^ gets dismutated into hydrogen peroxide (H_2_O_2_) and oxygen (O_2_) either spontaneously or due to the action of superoxide dismutase (SOD) enzymes^5^. The H_2_O_2_ then reacts with iron (Fe^2+^) via Fenton reaction to yield hydroxyl radicals (HO^−^) or is converted into hypochlorous acid (HClO) by a special enzyme called myeloperoxidase which is found in neutrophils^6^. Additionally, O_2_^−^ combines with nitric oxide (NO), synthesized by inducible nitric oxide synthase (iNOS), in macrophages to generate peroxynitrite (ONOO^−^)^4, 7^. All the above mentioned ROS (O_2_^−^, H_2_O_2_, HO^−^, ONOO^−^ and HClO) are highly toxic and have the ability to oxidize macromolecules such as proteins, lipids and DNA^5^. Further, the ROS have the ability to produce organic hydroperoxides in secondary reactions which can mediate additional oxidative damage^5^. Therefore, the ROS within phagocytes are considered as arsenals against invading microbes.

Bacterial pathogens, in general, have developed mechanisms to combat ROS generated by host phagocytes or by their own metabolism. Primarily, these mechanisms involve antioxidant enzymes such as SOD, catalase and peroxiredoxin, and these enzymes detoxify the ROS by acting upon them^8–10^. While SODs catalyze the dismutation of O_2_^−^ into H_2_O_2_ and O_2_ as mentioned earlier, catalases reduce the H_2_O_2_ further into H_2_O and O_2_. Conversely, peroxiredoxins reduce the organic peroxides (ROOH) into their corresponding alcohols, although they have the ability to reduce H_2_O_2_ into H_2_O and O_2_^11, 12^. When bacteria encounter stress due to a specific ROS, expression levels of the enzymes associated with the detoxification of the ROS is altered and this process is generally known as oxidative stress response^13, 14^. For instance, if the bacterium faces stress due to H_2_O_2_, then catalase and alkyl hydroperoxide reductase C (AhpC) levels are increased. To achieve this, the genes encoding these enzymes are regulated at transcriptional levels by oxidative stress response regulators. Each of these regulators senses a specific oxidant and responds to it by activating or derepressing a specific set of genes under its control, which are otherwise known as ‘regulon’ genes. In bacteria, SoxRS, OxyR, PerR and OhrR are some of the commonly found oxidative stress response regulators^5, 13, 15–17^. Whereas SoxRS responds to superoxide stress, the other regulators respond to either peroxide (OxyR and PerR) or organic hydroperoxide stress (OhrR)^5^. Interestingly, the presence or absence of any of these regulators as well as their numbers differs extensively from species to species, a phenomenon probably associated with the evolution of bacterial species.

The oxidative stress response regulator OhrR is part of the MarR family of bacterial regulators and it exists only in a select number of Gram positive and Gram negative bacterial species^5, 18^. It is closely related to the other MarR family of transcriptional regulators such as OspR of *Pseudomonas aeruginosa*, and MgrA and SarZ of *Staphylococcus aureus*. However, unlike OspR, SarZ and MgrA, which are global regulators, OhrR primarily regulates the expression of organic hydroperoxide reductase (Ohr), an enzyme of the OsmC/Ohr family^19–21^, although recent studies have reported that there are other proteins under the control of OhrR in certain species^22, 23^. In most bacterial species, OhrR binds to the promoter region of the *ohr* gene and to its own promoter region, and represses the expression of Ohr and OhrR during unstressed conditions^5, 24^. It releases its binding from the promoters only when it senses stress due to organic hydroperoxides and this release is accompanied by the expression of both Ohr and OhrR. In fact, during organic hydroperoxide stress, OhrR not only senses the stress but also gets oxidized at its cysteine residue(s), thus leading to changes in its structural configuration which eventually renders its release from the promoter region^5^. Although OhrR is classified, as one cysteine OhrR as in *Bacillus subtilis* ^21, 25, 26^ and two cysteine OhrR as in *Xanthomonas campesteris* ^19, 27^, based on the cysteine residues involvement in sensing organic hydroperoxide, both types of OhrR seem to play functionally similar roles, which is to repress the expression of *ohr* ^5^.

*Mycobacterium smegmatis* is a fast growing environmental mycobacterium which rarely infects humans. However, it has the ability to survive inside macrophages *in vitro* for a limited period of time^28^. Because of this attribute, this species has been used as a surrogate model for intracellular mycobacteria such as *M*. *tuberculosis*, *M*. *leprae* and other related species to understand their pathogenic aspects^29–32^. We have previously reported that deletion of the *ohrR* gene in *M*. *smegmatis* leads to the upregulation of Ohr expression and that the *ohrR* mutant strain (MSΔohrR) is more resistant to organic hydroperoxide stress as well as to the anti-tuberculosis drug, isoniazid^32^. We also noticed that the MSΔohrR strain had an enhanced survival in murine macrophages as compared to wild type *M*. *smegmatis*. Here, we report that OhrR can sense and respond to intracellular organic hydroperoxide stress and that this requires the N-terminal cysteine residue. Also, we show that this response of OhrR is not affected by intracellular superoxide and nitric oxide levels. To our knowledge, this is the first report demonstrating the ability of OhrR in sensing intracellular organic hydroperoxide stress.

## Results

### *ohr* driven *gfpuv* expression is repressed by OhrR

**Figure 1:**
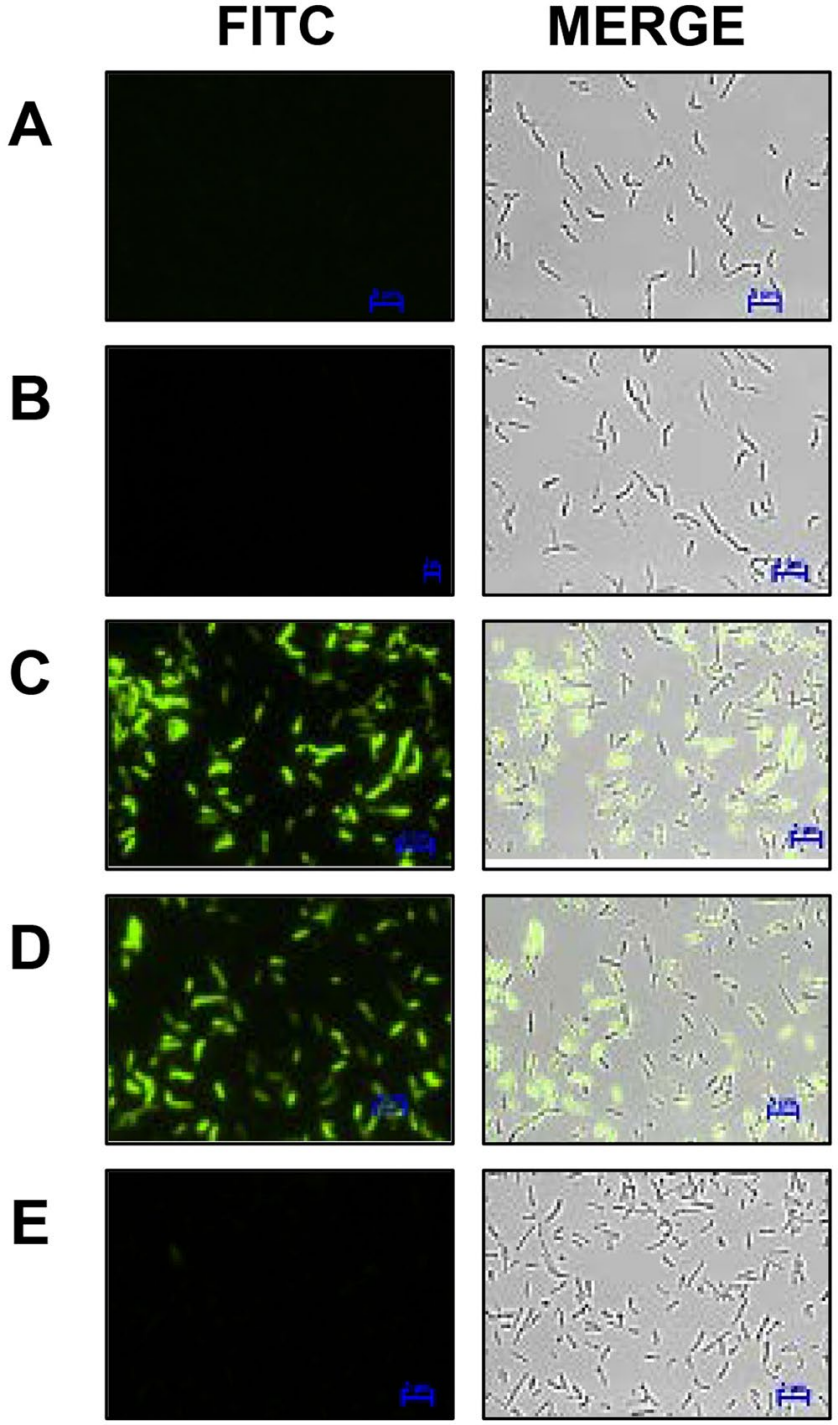
Examination of GFP expression in *M*. *smegmatis* strains by fluorescent microscopy. Green fluorescence in bacteria was detected using FITC filter in Nikon TiE Inverted Fluorescence Microscope with 60X objective. (**A–E**) *M*. *smegmatis* strains. Wild-type strain (**A**), MSohRE (**B**), MSOHG1 (**C**), MSOHG2 (**D**) and MSOHG3 (**E**). MSohRE is a plasmid control. Strains MSOHG1, MSOHG2 and MSOHG3 carry *pohrR*-*gfpuv*, *pohr*-*gfpuv and ohrR*-*pohr*-*gfpuv* fusions, respectively. FITC, images obtained under FITC filter; MERGE, FITC and DIC images merged together.

**Figure 2:**
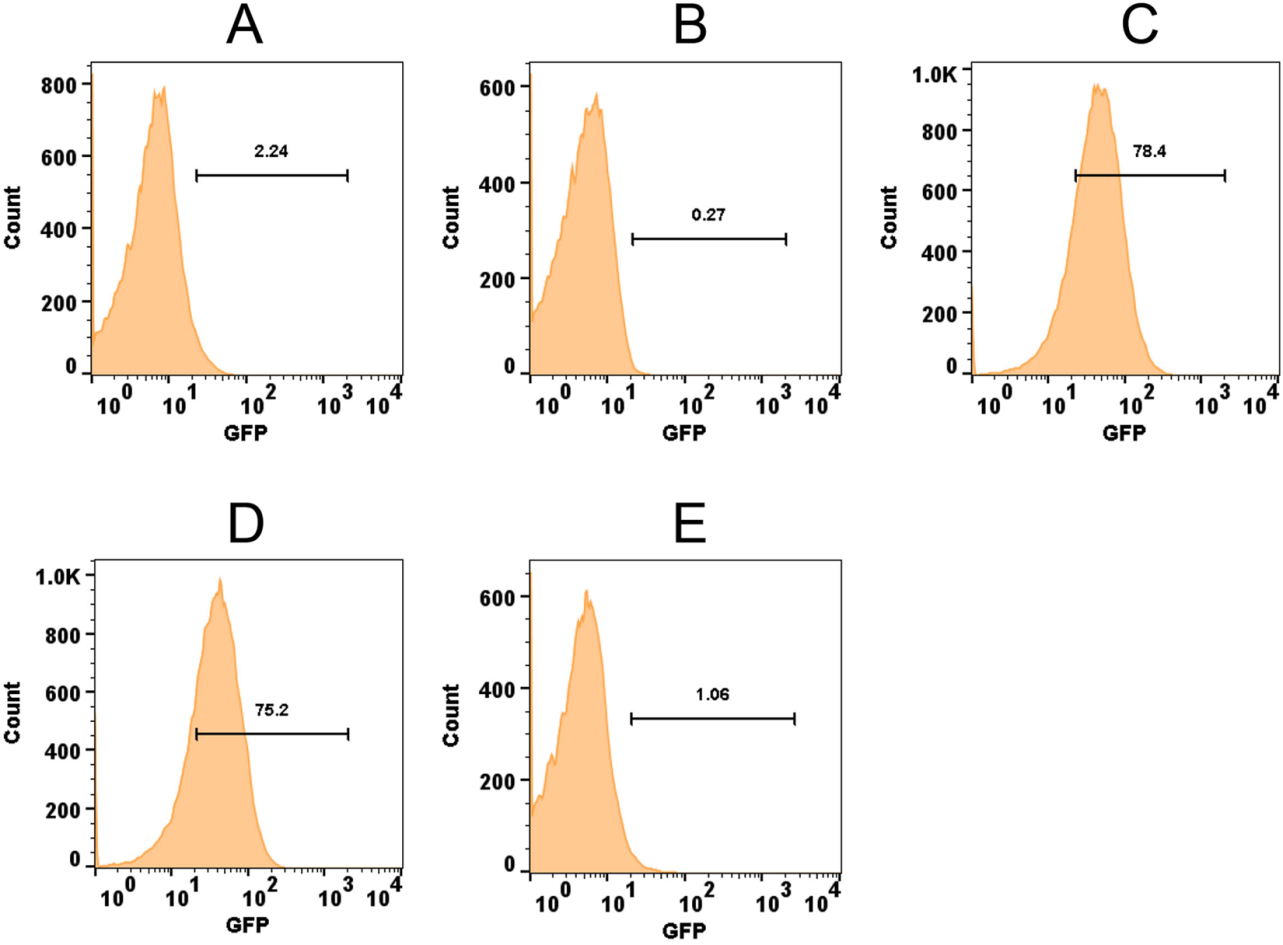
Examination of GFP expression in *M*. *smegmatis* strains by flow cytometry. Flow analysis was performed in BD FACSAriaII using excitation and emission wavelengths of 395 nm and 509 nm, respectively. (**A–E**) *M*. *smegmatis* strains. Wild-type (**A**), MSohRE (**B**), MSOHG1 (**C**), MSOHG2 (**D**) and MSOHG3 (**E**). MSohRE is a plasmid control. Strains MSOHG1, MSOHG2 and MSOHG3 carry *pohrR*-*gfpuv*, *pohr*-*gfpuv and ohrR*-*pohr*-*gfpuv* fusions, respectively.

It was previously noted that *ohr* is divergently transcribed from *ohrR* in *M*. *smegmatis* and that *ohr* expression is tightly repressed by OhrR^32^. To prove this experimentally with a reporter system and also to demonstrate OhrR’s ability to sense intracellular organic hydroperoxide stress, we constructed three plasmids: pMOHGFP1, pMOHGFP2 and pMOHGFP3 with a GFP reporter system as depicted in Figure S1. These plasmids were transformed into wild-type *M*. *smegmatis* and the resulting strains MSOHG1, MSOHG2 and MSOHG3 were examined by fluorescent microscopy for the expression of GFP (Fig. 1). Although bacteria expressing GFP were readily observed in both MSOHG1 and MSOHG2 strains, which carry *gfpuv* transcriptionally fused with *pohrR* and *pohr* promoters, respectively, without an intact *ohrR* gene (Fig. 1C and D), no GFP expressing bacteria were observed from the strain MSOHG3 which bears *gfpuv* transcriptionally fused with *pohr* in the presence of an intact *ohrR* gene (Fig. 1E). In fact, the MSOHG3 bacteria behaved, in terms of GFP expression, similar to that of bacteria from wild type *M*. *smegmatis* strain (Fig. 1A) and the *M*. *smegmatis* strain MSohRE (plasmid control without *gfpuv*; Fig. 1B). As observed with microscopy, flow cytometry analyses of the bacteria from these strains (Fig. 2) revealed no GFP expressing bacteria in MSOHG3 strain and over 70% of bacteria expressing GFP in MSOHG1 and MSOHG2 strains. These results suggest that OhrR produced by the plasmid borne *ohrR* represses *ohr* expression, and consequently the GFP fluorescence in MSOHG3. It should be noted that MSOHG1 and MSOHG2 still have a chromosomal copy of *ohrR* and its product could have exerted its effect on the *pohrR* and *pohr* promoters in the plasmids of these strains. Despite this, the expression of GFP in these strains is an indication that the OhrR produced by the chromosomal copy of *ohrR* was not sufficient to completely repress the transcription of these promoters since they are based on multi-copy plasmids.

### *pohr*-*gfpuv* expression can be induced

**Figure 3:**
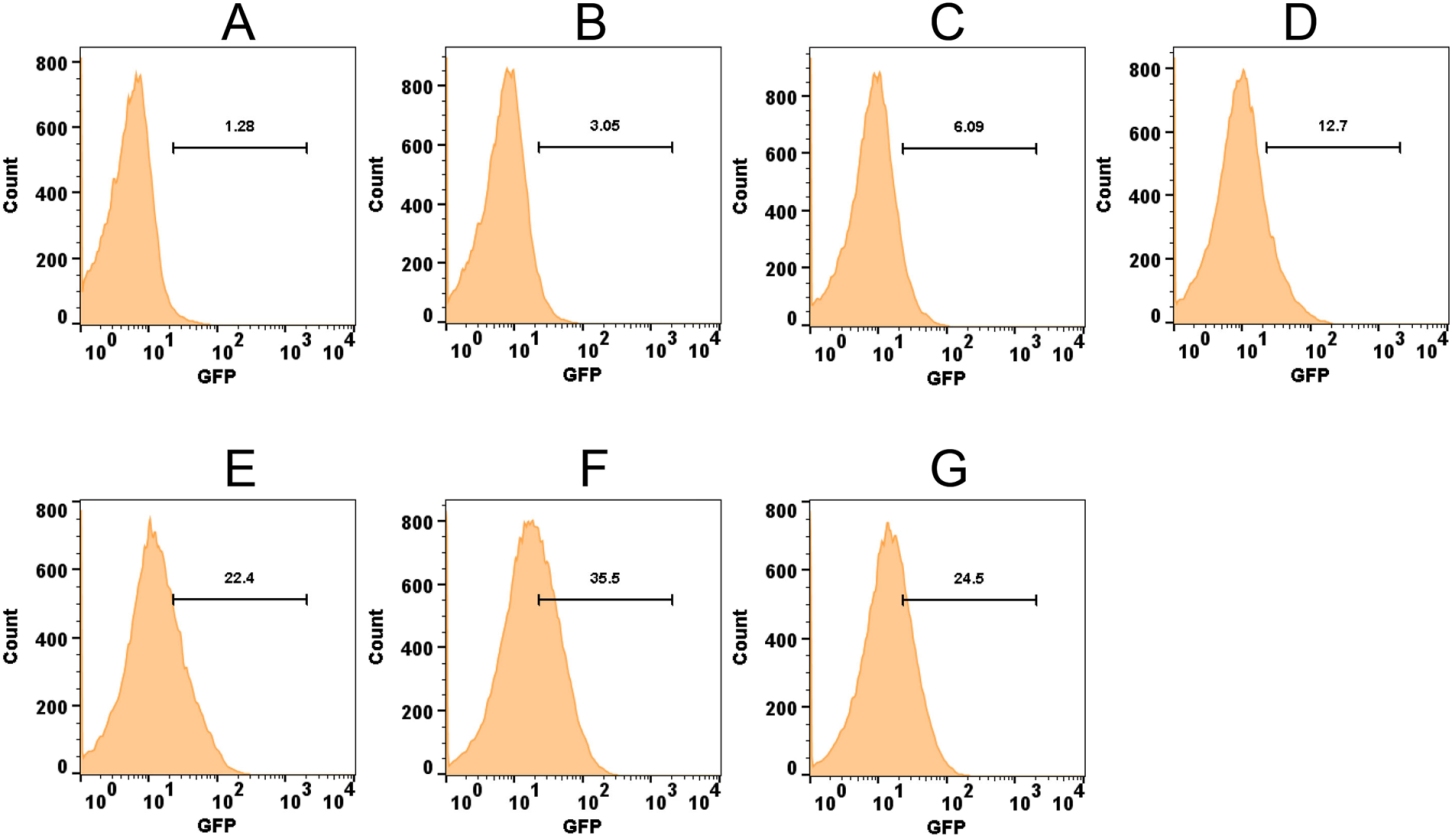
Induction of GFP in *M*. *smegmatis* strain MSOHG3 by different concentrations of *t*-BHP. MSOHG3 carrying *ohrR*-*pohr*-*gfpuv* fusion was treated with different concentrations of *t*-BHP and incubated at 37 °C for 2 h. Flow analysis was performed in BD FACSAriaII using excitation and emission wavelengths of 395 nm and 509 nm, respectively. (**A**) un-induced control bacteria; (**B–G**) bacteria induced with 25, 50, 100, 250, 500 and 1000 µM *t*-BHP, respectively.

Since Ohr expression is inducible in *M*. *smegmatis* cells by treating with cumene hydroperoxide (CHP) or *t*-butyl hydroperoxide (*t*-BHP)^32^, we speculated that *pohr* driven *gfpuv* expression in MSOHG3 strain could also be induced by these chemicals. Thus, we tested MSOHG3 for the induction of GFP expression by CHP and *t*-BHP along with H_2_O_2_, superoxide generator menadione, and sodium hypochlorite (NaOCl) for control purposes. Although CHP and *t*-BHP induced GFP in this strain (Fig. S2), other oxidants showed no induction of GFP expression despite testing at different concentrations (data not shown). However, flow cytometry and fluorescent microscopy (Fig. 3 and Fig. S3) analyses revealed that GFP expression in this strain was upregulated by *t*-BHP in a dose dependent manner from 25 µM onwards. The maximum induction (36% of cells) was noticed at a concentration of 500 µM *t*-BHP and any concentration beyond that level (1 mM) showed reduction in GFP expression (20% of cells), indicating higher concentration of *t*-BHP has an inhibitory effect.

**Figure 4:**
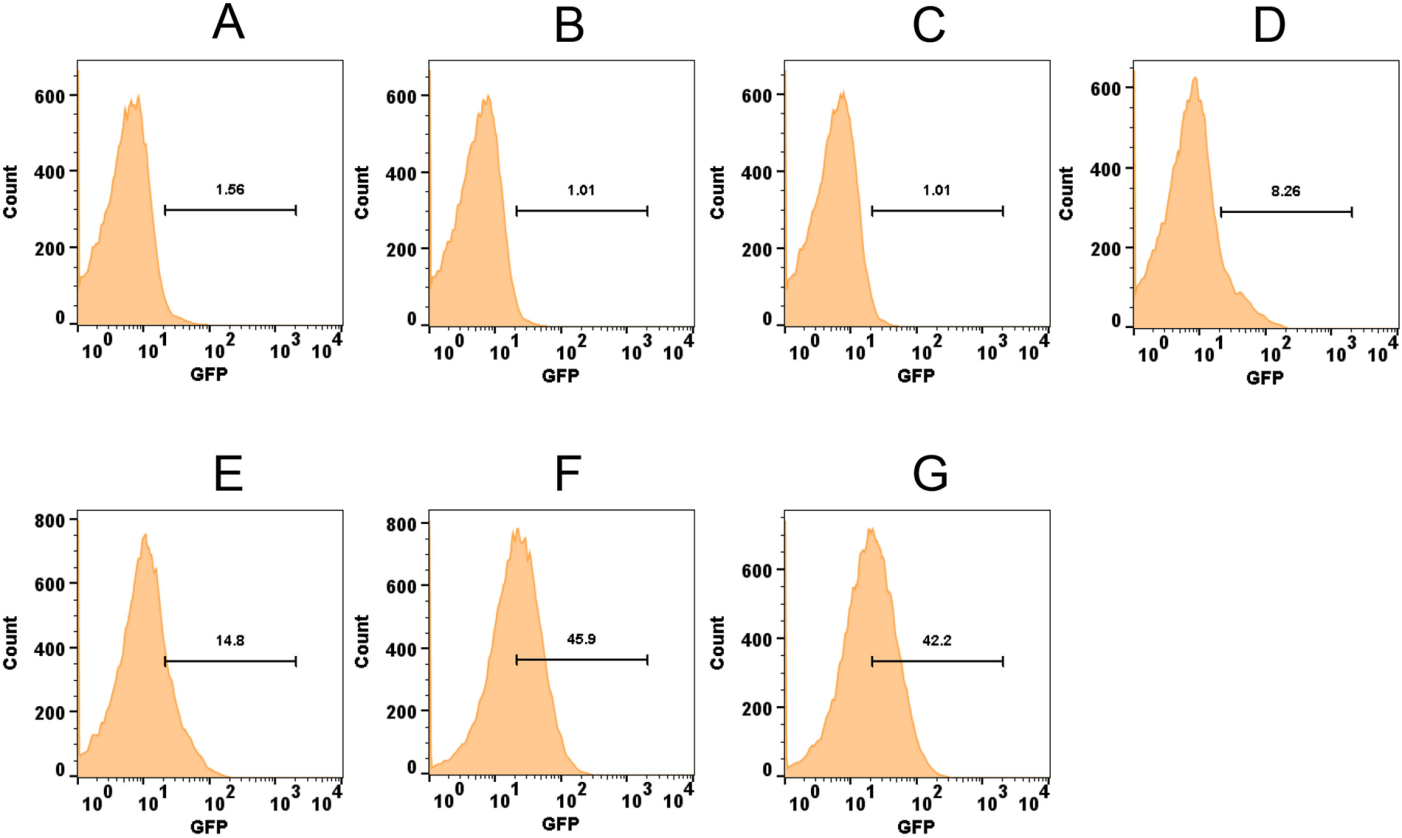
Induction of GFP in *M*. *smegmatis* strain MSOHG3 exposed to *t*-BHP for different time points. MSOHG3 carrying *ohrR*-*pohr*-*gfpuv* fusion was exposed to 100 µM of *t*-BHP for different time periods at 37 °C. Flow analysis was performed in BD FACSAriaII using excitation and emission wavelengths of 395 nm and 509 nm, respectively. (**A**) un-induced control bacteria; (**B–G**) bacteria exposed to *t*-BHP for 0, 15, 30, 60, 120, 240 minutes, respectively.

Further, to understand whether exposure time to organic hydroperoxides has any role in the induction of *ohr*, we exposed the MSOHG3 strain to a relatively lower concentration (100 µM) of *t*-BHP and assessed the GFP expression at different time points. This revealed that GFP expression (Fig. 4 and Fig. S4) could be induced to measurable levels (8% cells) after 30 min of post-exposure (Fig. 4D). Although the expression continued to show incremental increase (45% cells at 2 h) up to 2 h of post-exposure, longer exposure of up to 4 h did not show any additional increase in GFP expression (42% cells; Fig. 4G), indicating that induction reached saturation levels by 2 h. Collectively, these results indicated that OhrR tightly regulates *ohr* expression and this can be derepressed by organic hydroperoxides in a concentration and time dependent manner.

### Intracellular bacteria induce *pohr*-*gfpuv*

**Figure 5:**
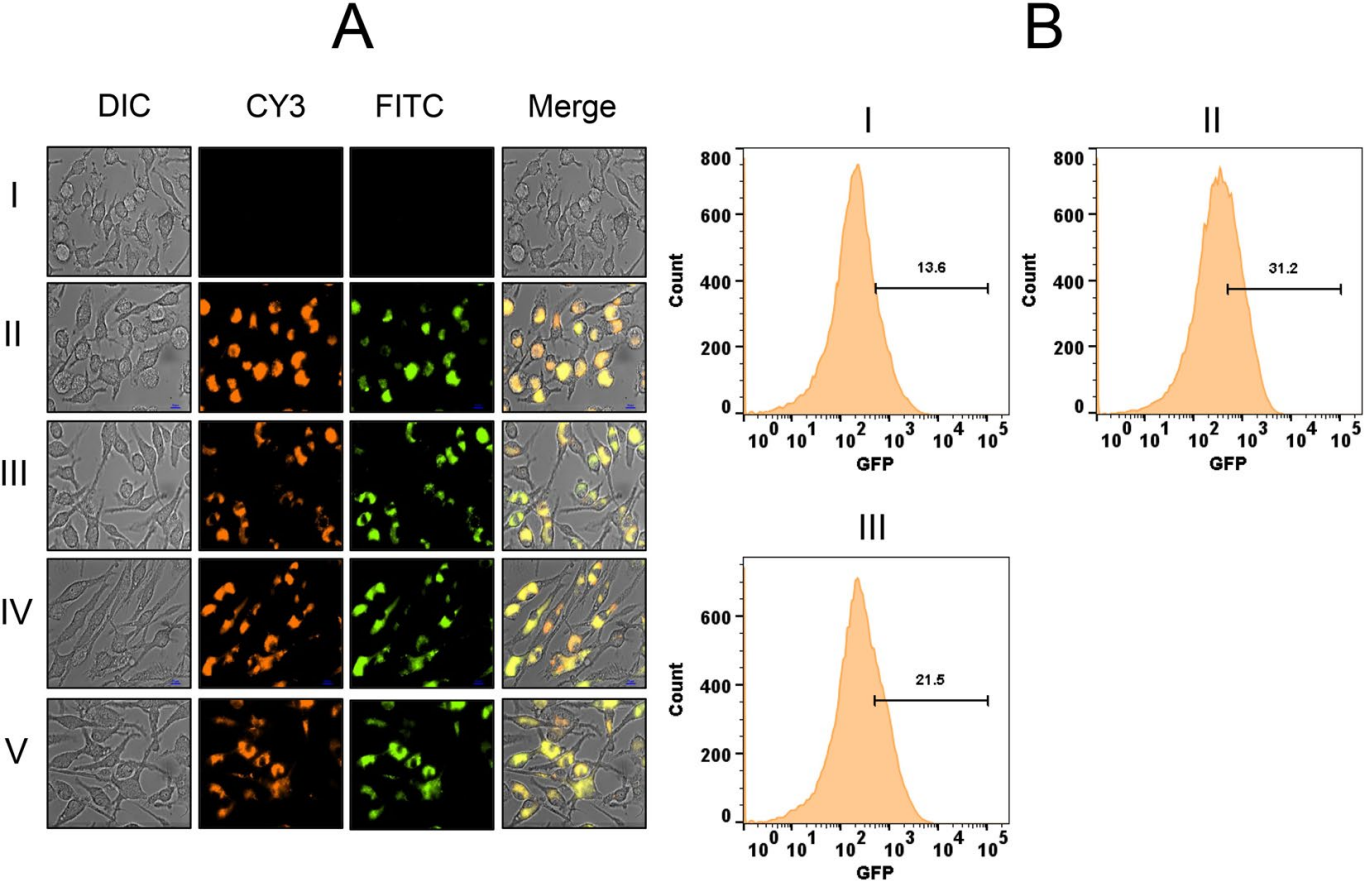
Induction of GFP in *M*. *smegmatis* MSOHG4 strain by intracellular organic hydroperoxides of macrophages. (**A**) GFP expression assessed by microscopy. BMDM cells grown on glass coverslips were infected with *M*. *smegmatis* MSOHG4 bearing *ohrR*-*pohr*-*gfpuv* and *phsp60*-*rfp* fusions for 2, 4, 12 and 24 h at 37 °C. After washing, the coverslips were examined under Nikon TiE Inverted Fluorescence Microscope using 60X objective. DIC, Cy3 and FITC indicate images obtained using these filters. ‘Merge’ indicates the merged images of DIC, CY3 and FITC. I, Images of uninfected BMDM, and II–V, images of BMDM infected with MSOHG4 for 2, 4, 12 and 24 h of infection, respectively. (**B**) GFP expression assessed by flow cytometry. BMDM cells grown on culture dishes were infected with *M*. *smegmatis* wild type (I), MSOHG2 (II) and MSOHG3 (III) for 4 h at 37 °C. After washing, cells were processed for flow cytometry. Flow analysis was performed in BD FACSAriaII using excitation and emission wavelengths of 395 nm and 509 nm, respectively.

Next, we investigated whether intracellular organic hydroperoxides can induce *pohr*-*gfpuv*. To determine this, we transformed the plasmid pMOHGFP4 (Fig. S5) in the *M*. *smegmatis* strain MSΔohr-ohrR which lacks genes for both Ohr and OhrR. The resulting MSOHG4 strain revealed, upon examination by fluorescent microscopy and flow cytometry, that it has the ability to express RFP constitutively and GFP upon induction with *t*-BHP (Fig. S6). The constitutive expression of RFP by the bacteria of this strain allowed the co-localization of GFP induced bacteria upon entry into macrophages. Infection of BMDM with MSOHG4 revealed that expression of GFP in bacteria, due to the induction of *ohr*, could be visualized after 4 h post-infection (Fig. 5A-II), a standard time point that has been used for mycobacterial infection of macrophages. However, examination of the BMDM cells infected with MSOHG4 for different points, from 2 h to 24 h post-infection, showed that GFP expression in the strain could be visualized even before 2 h. However, no discernible differences in fluorescence levels could be visualized by infecting macrophages for longer period after 24 h (Fig. 5A-II to V). RAW264.7 cells infected with MSOHG4 also displayed a similar profile (Fig. S7-I and II). In addition, activation status of BMDM, that is unactivated or activated in the presence of IFN-γ also had no influence on the intracellular fluorescence levels of MSOHG4 after 4 h and 24 h (data not shown). Furthermore, infection of BMDM with MSOHG3, which lacks the *rfp* gene, in parallel also resulted in similar results, indicating that a longer exposure to the macrophage environment has only limited impact on Ohr based GFP expression. To understand whether this was due to any limitation in detecting different levels of GFP fluorescence in infected cells, we infected BMDM with wild-type *M*. *smegmatis*, MSOHG2 and MSOHG3 strains and analyzed the cells for GFP fluorescence by flow cytometry. The results (Fig. 5B) showed different levels of intracellular GFP expressing bacteria for the MSOHG2 (31% cells) and MSOHG3 (21% cells) strains, indicating that flow cytometry had no limitation in detecting the differences in fluorescence.

### Phox generated O_2_^−^ has no effect on *pohr*-*gfpuv* induction

**Figure 6:**
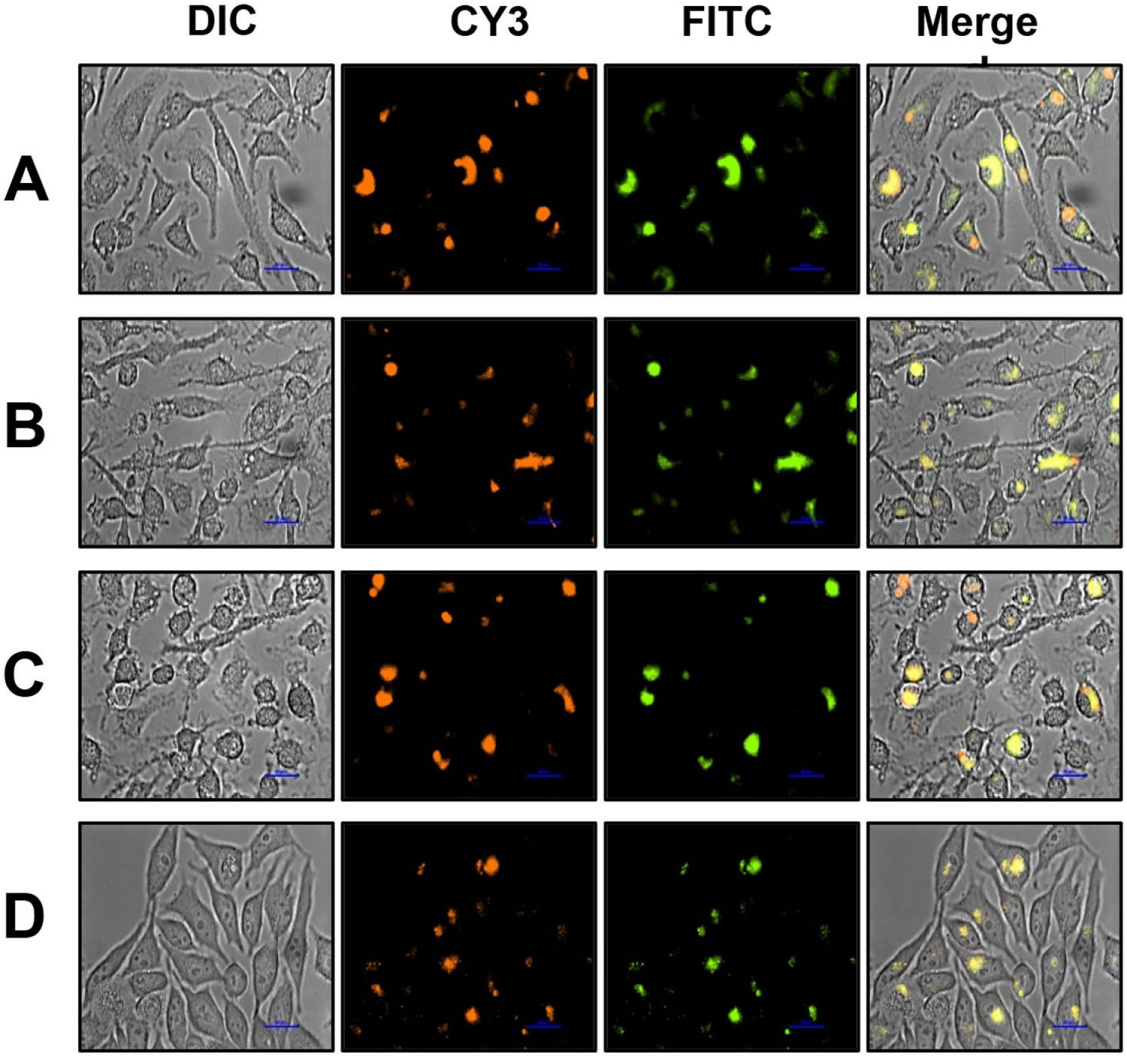
Induction of GFP in *M*. *smegmatis* MSOHG4 strain by intracellular organic hydroperxoides of mutant macrophages and epithelial cells. BMDM cells from *Phox* ^−/−^, *iNOS* ^−/−^, *MPO* ^−/−^ mice and HeLa epithelial cells were grown on glass coverslips and infected with *M*. *smegmatis* MSOHG4 bearing *ohrR*-*pohr*-*gfpuv* and *phsp60*-*rfp* fusions for 4 h at 37 °C. After washing, the coverslips were examined under Nikon TiE Inverted Fluorescence Microscope with 60X objective. DIC, Cy3 and FITC indicate images obtained using these filters. MERGE indicates the merged images of DIC, CY3 and FITC. A, BMDM from *Phox* ^−/−^ mice; B, BMDM from *iNOS* ^−/−^ mice; *C*, BMDM from *MPO* ^−/−^ mice; D, HeLa cells.

As noted before, phagocytes have special enzymes to produce O_2_^**−**^, NO and HClO and these ROS can react with organic compounds within macrophages to produce organic hydroperoxides. To determine the roles of these ROS in organic hydroperoxides production within macrophages and their influence on OhrR, we infected BMDM obtained from mice lacking in phagocyte oxidase (*PhoX* ^−/−^) or inducible nitric oxide synthase (*iNOS* ^−/−^) or myeloperoxidase (*MPO* ^−/−^) with MSOHG4 strain. Results shown in Fig. 6A–C reveal that all these cells could induce similar levels of GFP expression in MSOHG4, indicating that OhrR oxidation within macrophages is not dependent upon of the oxidative radicals generated by these enzymes. Although this was unexpected and surprising, because these enzymes are considered as the major source for ROS production within macrophages, this indicated the possibility that non-phagocytic cells might also induce Ohr in the intracellular environment. To assess this, we infected HeLa cells with MSOHG4 strain and observed them under the microscope. As can be seen in Fig. 6D, we could see GFP expressing bacteria in these cells, although their numbers were quite less. These results suggest that all eukaryotic cells have some basal levels of organic hydroperoxides and they can oxidize OhrR to induce Ohr expression.

### Cysteine residue of OhrR is critical for the induction of *pohr*-*gfpuv*

**Figure 7:**
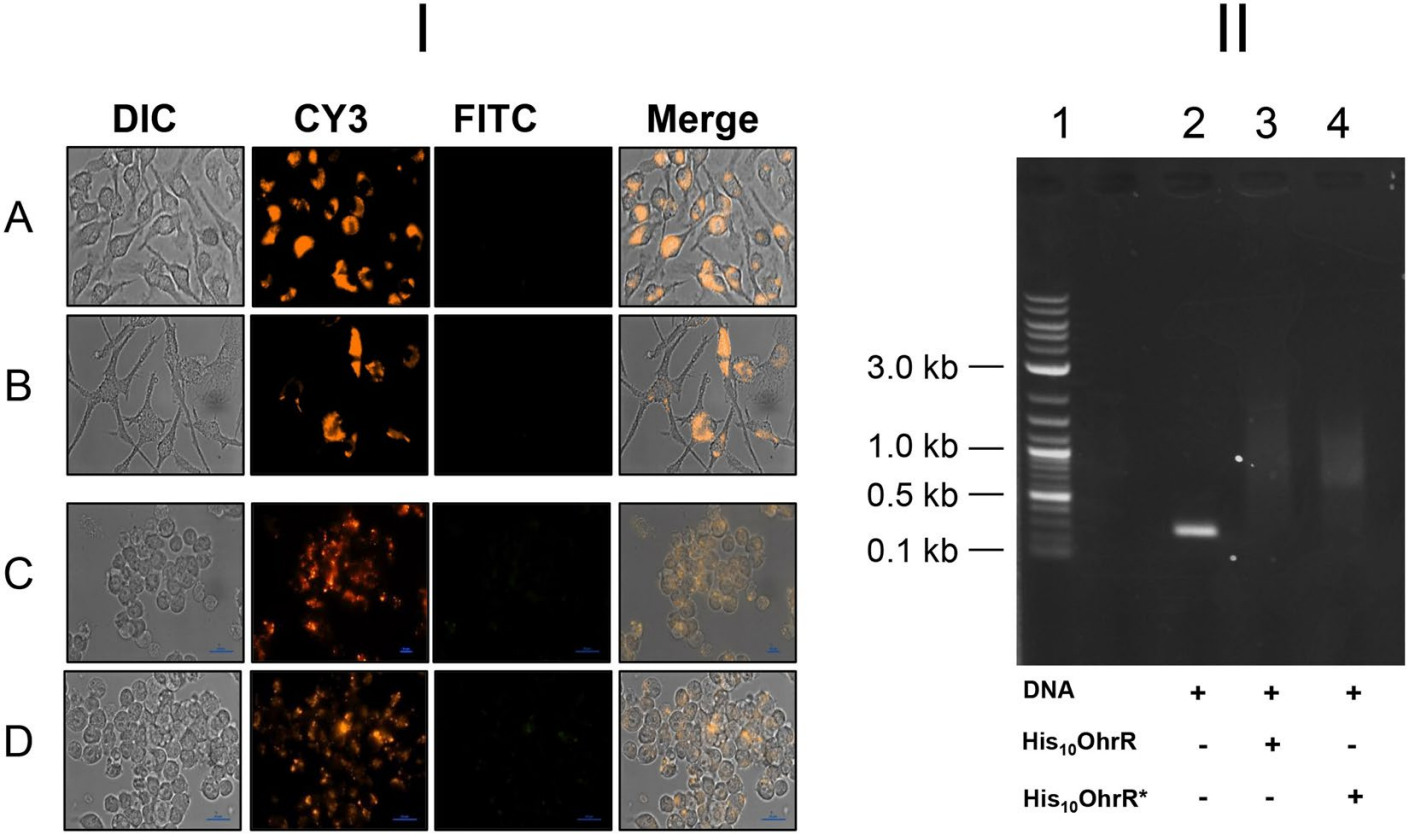
*M*. *smegmatis* strain MSOHG5 bearing Cys mutated OhrR shows no GFP induction. I. MSOHG5 in the intracellular environment. BMDM (**A** and **B**) and RAW264.7 (**C** and **D**) cells grown on glass coverslips were infected with *M*. *smegmatis* MSOHG5 bearing ohrR*-*pohr*-*gfpuv* and *phsp60*-*rfp* fusions for 4 h (**A** and **C**) and 24 h (**B** and **D**) at 37 °C. After washing, the coverslips were examined under Nikon TiE Inverted Fluorescence *M*icroscope using 60X objective. DIC, Cy3 and FITC indicate the images obtained using these filters. MERGE indicates merged DIC, CY3 and FITC images. II. EMSA showing the interaction of recombinant Cys mutated OhrR protein of *M*. *smegmatis* with *ohr*-*ohrR* intergenic region. EMSA was performed as described in the methods section. Lane 1, DNA Marker; Lane 2, *ohr*-*ohrR* intergenic region; Lane 3, *ohr*-*ohrR* intergenic region with non-mutated OhrR (MSHis_10_OhrR); Lane 4, *ohr*-*ohrR* intergenic region with Cys mutated OhrR (MSHis_10_OhrR*).

It has previously been reported that cysteine (Cys) residues of OhrR are the sensors for organic hydroperoxide stress, although the number of Cys residues involved in sensing organic peroxides vary between species^5, 20, 21^. The OhrR of *M*. *smegmatis* has only one Cys residue located at the amino acid position 13 in the N-terminal region and we hypothesized that modification of this Cys13 into some other amino acid will fail to sense the organic hydroperoxide stress within macrophages, and as a consequence *pohr*-*gfpuv* expression will not be induced. To prove this, we generated the plasmid pMOHGFP5 in which the *ohrR* gene is modified to encode arginine (Arg) in the place of Cys13. This plasmid was transformed into MSΔohr-ohrR strain and the resulting strain MSOHG5 was examined for the induction of GFP expression (*pohr*-*gfpuv*) by *t*-BHP *in vitro* and by intracellular peroxides by infecting BMDM. Results presented in Fig. 7-I reveal that GFP expression was not induced in these bacteria by *t*-BHP *in vitro* and by organic hydroperoxides in the intracellular environment. This provided convincing evidence that OhrR of *M*. *smegmatis* indeed senses and responds to intracellular organic hydroperoxide stress. Further, since non-induction of GFP is an indication of the strong binding of mutated OhrR to the promoter region of *ohr*, we investigated the binding ability of cysteine mutated OhrR *in vitro* in EMSA. The results (Fig. 7-II) show that both unmutated (His_10_OhrR) and mutated (His_10_OhrR*) OhrR have the ability to bind with the promoter region of *ohr*, indicating that the mutated OhrR in the strain MSOHG5 could still bind to the *ohr* promoter region and suppress its expression but was unable to get oxidized by organic hydroperoxides.

## Discussion

In this study, we generated GFP expressing *M*. *smegmatis* strains by transcriptionally fusing the promoter regions of *ohr* and *ohrR* to *gfpuv*. Initial *in vitro* studies demonstrated that *M*. *smegmatis* bearing the *pohr*-*gfpuv* fusion construct exhibits elevated expression of GFP in the absence of *ohrR* gene (MSOHG2 strain) but no expression in the presence of functional *ohrR* gene (MSOHG3 strain). However, it was noticed that the GFP expression in the latter strain (MSOHG3) could be induced by organic hydroperoxides (*t*-BHP and CHP) in a time and dose dependent manner. These observations are consistent with our previous findings that *ohr* is under the tight control of OhrR in *M*. *smgematis* and its expression occurs only if OhrR is oxidized^32^. Additionally, these results corroborate with similar observations made in other species of bacteria like *X*. *campestris* ^19^, *Bacillus subtilis* ^33^, *Pseudomonas aeruginosa* ^34^, *Streptomyces coelicolor* ^35^, *Agrobacterium tumefaciens* ^36^, *Sinorhizobium meliloti* ^37^ and *Chromobacterium violaceum* ^27^. Further, the observation that the strain *M*. *smegmatis* MSOHG1, bearing *pohrR*-*gfpuv* but lacking the gene *ohrR*, expresses GFP similar to the levels of its counterpart MSOHG2 (*pohr*-*gfpuv*) without any induction, provides evidence that OhrR of *M*. *smegmatis* regulates its own expression, a phenomenon also noticed in several other bacteria^5^.

Notably, in addition to *in vitro* induction by *t*-BHP, GFP expression in the strain MSOHG3 and its parallel strain MSOHG4, which carry *ohr*-*gfpuv* fusion construct with a functional *ohrR* gene, is induced by the intracellular environment of macrophages like RAW264.7 and BMDM and to some extent by epithelial cells. This suggests that OhrR of *M*. *smegmatis* senses and gets oxidized by the intracellular organic hydroperoxides and the intracellular environment within the phagosomes has copious amounts of organic hydroperoxides. The latter is very significant because the amount of organic hydroperoxides required to derepress *ohr* within that time period, based on our *in vitro* study, is about 25 µm *t*-BHP. This observation has implications on the pathogenic mechanisms of bacterial pathogens that carry intact *ohr/ohrR* genes like *Brucella abortus* ^38^. It is possible that OhrR regulates *ohr* or other regulon genes in response to intracellular organic hydroperoxide stress in these species. Nonetheless, with respect to pathogenic mycobacteria, this possibility is limited because the major pathogenic mycobacteria like *M*. *tuberculosis*, *M*. *leprae* and *M*. *avium* are lacking in *ohr*/*ohrR* genes and sequence data in NCBI databases reveals that *ohr/ohrR* genes exist predominantly in non-pathogenic mycobacteria.

Interestingly, *pohr* driven GFP expression in MSOHG3 and MSOHG4 was not altered by the in the intracellular environment of macrophages which are deficient in Phox, iNOS and MPO enzymes. This implies that ROS resulting by the action of these enzymes contribute very little to the production of intracellular organic hydroperoxides that oxidize OhrR of the bacteria in the intracellular compartments. Although this explanation is easy, the molecules that oxidize the OhrR in the intracellular bacteria to induce the *pohr* based GFP expression remains an unanswered question. In this context, it should be pointed out that the natural organic hydroperoxides which oxidize OhrR in bacteria still remain elusive and several studies have employed only artificial compounds such as linoleic acid hydroperoxide (LAOOH), t-BHP, CHP and sodium hypochlorite (NaOCl) to induce Ohr in bacteria^5^. Very recently, a peroxynitrite generator, SIN-1, has been implicated in the induction of Ohr in *P*. *aeruginosa* ^39^. Thus, it is logical to assume that the organic hydroperoxides or molecules which oxidize OhrR within the intracellular compartments may bear structures similar to the inducers mentioned above. Alternatively, the possibility that lipid peroxides produced within the macrophages by eicosanoid metabolism^40^ or due to the oxidation of polyunsaturated fatty acids (PUFA)^41^ may also play a role in this process still exists.

In contrast to *ohr* of *M*. *smegmatis*, an OxyR dependent gene *ahpC*, which responds to hydrogen peroxide and alkyl hydroperoxide stress in enteric bacteria, has been observed to be affected by the absence of Phox enzyme in the intracellular environment^42^. In this study, a *Salmonella* strain carrying *ahpC*-*gfp* fusion was found to have only background levels of GFP expression inside the macrophages derived from the bone marrow of *gp91* phox^−/−^ mice, although it had higher levels of GFP expression inside macrophages derived from non-mutated mice (C57BL/6)^42^. This study also showed that mutant mice (*gp91 phox* ^−/−^) and non-mutated mice (C57BL/6) infected with the above *Salmonella* strain reflected the results obtained with macrophages, indicating that hydrogen peroxide or alkyl hydroperoxide levels in these macrophages are altered significantly by the absence of Phox enzyme. Although such a direct connection between the absence of Phox and generation of organic hydroperoxides is missing in our study, the fact that altered hydrogen peroxide levels in the gp91 phox^−/−^ macrophages has no effect on OhrR of *M*. *smegmatis* study indicates that OhrR is prone to oxidation only by organic hydroperoxides and not by other peroxides. Thus far, all bacterial OhrR are reported to get oxidized only by organic hydroperoxides and the only exception seems to be the OhrR of *Shewanella oneidensis* which gets oxidized by both organic hydroperoxides and hydrogen peroxide^22^.

We have shown that *pohr*-*gfpuv* expression is completely affected in strains bearing the *ohrR* gene which had a mutation to code for N-terminal Cys13 residue to Arg in both *in vitro* and in the intracellular environment. This reiterates the critical role of Cys residue(s) of OhrR in sensing organic hydroperoxides. It is now recognized that OhrR of single Cys category senses organic hydroperoxides by the N-terminal Cys residue (C15 in the case of *B*. *subtilis*) and OhrR of two Cys category senses organic hydroperoxides by both N-terminal and C-terminal Cys residues (C22 and C127 in the case of *X*. *campestris*), and sensing results in the oxidation of Cys residues^5, 20, 21^. In both cases, oxidation of N-terminal Cys by organic peroxides leads to the formation of sulfenic acid (C-SOH) initially. In one Cys OhrR, this gets further modified into Cys-S-S-R by mixing with reduced cellular thiols or into Cys-SN by reacting with amino group of a neighboring amino acid, and this second modification only inactivates the OhrR which leads to the derepression of *ohr*. On the other hand, in the two Cys OhrR, the Cys-SOH modification of the N-terminal Cys reacts with C-terminal Cys and form an intersubunit disulfide bond which leads to conformational changes and consequently the inactivation of OhrR.

Strikingly, Cys13 to Arg modification of OhrR of *M*. *smegmatis* in our study has affected only the sensing but not the binding of OhrR to the promoter region of *ohr*. This is evident from the repression of *pohr*-*gfpuv* expression in these constructs and also by the results of the EMSA (Fig. 7-II). Results identical to this situation have also been noticed in *X*. *campestris* where modification of Cys22 and Cys127 in OhrR to serine residues only affected the sensing of organic hydroperoxides and not its binding to the *ohr* promoter region^21^. Further, it should be noted that, although Cys and Arg belong to neutral and basic amino acids, respectively, substitution of Arg to Cys does not seem to affect the fold and function of OhrR of *M*. *smegmatis*.

In conclusion, we have shown that OhrR of *M*. *smegmatis* can be induced by intracellular organic hydroperoxide stress and the levels of intracellular organic hydroperoxides sensed by OhrR are not greatly altered by the absence of Phox, iNOS and MPO enzymes. Although the expression of GFP in *M*. *smegmatis* strain bearing *ohrR*-*pohr*-*gfpuv* fusion can be altered by exposing the bacteria to different concentrations of organic hydroperoxides, absence of significant changes in the intracellular environment limits the utility of OhrR based GFP reporter system in host-pathogen interactions studies of pathogenic mycobacteria. However, the fact that OhrR responds to the intracellular environment suggests that OhrR-Ohr components can exploited for the delivery specific peptides and proteins to the intracellular compartments.

## Methods

### Bacterial strains and growth conditions

*Escherichia coli* strains DH5-α and BL21 (DE3) were used to clone DNA fragments in plasmids and to overexpress recombinant proteins, respectively. They were grown in Luria-Bertani (LB) broth or LB agar with appropriate antibiotics (100 μg/ml ampicillin or 25 μg/ml kanamycin) at 37 °C. Mycobacterial species *M*. *smegmatis* (*mc* ^*2*^ *155*) was grown at 37 °C in Middlebrook 7H9 broth or 7H10 agar plates supplemented with 100 ml per liter of albumin dextrose complex (ADC; 5 g bovine serum albumin, 2 g D-dextrose and 0.85 g of NaCl per 100 ml), 0.2% glycerol and 0.05% Tween 80 (TW). *M*. *smegmatis* strains harboring plasmids or antibiotic resistance genes were grown in 7H9 or 7H10 medium containing kanamycin 25 μg/ml or hygromycin 50 μg/ml. The bacterial strains used in the study are given in Table 1.

**Table 1:** Bacterial strains and plasmids used in this study.

Strain or plasmid	Characteristics	Source or reference
***E***. ***coli***		
DH5-α	*lacZ∆M15 recA1*	Invitrogen
BL21 (DE3)	DE3 λ prophage carrying the T7 RNA polymerase gene and lacI^q^	Invitrogen
***M***. ***smegmatis***		
*mc* ^*2*^ *155* (MSWt)	Wild type	ATCC #27294
MSohrE	*M*. *smegmatis* wild type strain harboring pMVMSOHR2. This plasmid a derivative of pMV206 which bears *ohr* gene and its promoter region of *M*. *smegmatis*.	32
MSΔohr-ohrR	*M*. *smegmatis ohr*-*ohrR* disrupted with hyg^r^ marker	32
MSOHG1	*M*. *smegmatis mc* ^*2*^ *155* carrying plasmid pMOHGFP1	This study
MSOHG2	*M*. *smegmatis mc* ^*2*^ *155* carrying plasmid pMOHGFP2	This study
MSOHG3	*M*. *smegmatis mc* ^*2*^ *155* carrying plasmid pMOHGFP3	This study
MSOHG4	MSΔohr-ohrR carrying plasmid pMOHGFP4	This study
MSOHG5	MSΔohr-ohrR carrying plasmid pMOHGFP5	This study
**Plasmids**		
pCR2.1	TA cloning vector Amp^r^Km^r^	Invitrogen
pET16b	Overexpression vector carrying an N-terminal His-Tag	Novagen
pGFPUV	*E*. *coli* plasmid bearing *gfpuv* gene Amp^r^	Clontech/Invitrogen
pMV206	*E*. *coli*- Mycobacteria shuttle vector	43
pMSOHR	pCR2.1 vector harboring 2053 bp *ohr/ohrR* region of *M*. *smegmatis*	32
pEOHUV1	pGFPUV carrying 400 bp DNA fragment containing *ohr*-*ohrR* intergenic region, and creating the fusion of *pohrR*-*gfpuv*	This study
PEOHUV2	pGFPUV carrying 400 bp DNA fragment containing *ohr*-*ohrR* intergenic region, and creating the fusion of *pohr*-*gfpuv*	This study
pEOHUV3	pGFPUV carrying 1270 bp DNA fragment containing full *ohrR gene with* intergenic region, and creating the fusion of *pohr*-*gfpuv*	This study
pEOHUV3m	pGFPUV carrying 1270 bp DNA fragment containing full mutated ohrR* gene with intergenic region, and creating the fusion of *ohr*-*gfpuv*	This study
pMOHGFP1	pMV206 carrying *pohrR*-*gfpuv* fusion	This study
pMOHGFP2	pMV206 carrying *pohr*-*gfpuv* fusion	This study
pMOHGFP3	pMV206 carrying *ohrR*-*pohr*-*gfpuv* fusion	This study
pMOHGFP4	pMVRFP carrying *ohrR*-*pohr*-*gfpuv* fusion	This study
pMOHGFP5	pMVRFP carrying ohrR*-*pohr*-*gfpuv* fusion (mutated *ohrR*)	This study
p16MSOHRREX2	pET16b containing *M*. smegmatis ohrR* (mutated ohrR) coding region	This study

### Plasmid construction

Several plasmids were constructed to express GFP in mycobacteria either using the *ohr* promoter (*pohr*) or *ohrR* promoter (*pohrR*). As can be seen in Figure S1, the *ohr* (*MSMEG_447*) and *ohrR* (*MSMEG_448*) genes are divergently transcribed from each other and the intergenic region contains the promoters for both genes. The intergenic region containing partial coding regions of *ohr* and *ohrR* (400 bp) was obtained by digesting the plasmid pMSOHR^32^ with NcoI and StuI. This fragment was blunt ended by Klenow treatment and ligated to XbaI cut and blunt ended pGPFUV (Clontech/Invitrogen) plasmid. This resulted into plasmids pEOHUV1 and pEOHUV2 bearing *pohrR*-*gfpuv* and *pohr*-*gfpuv* transcriptional fusions, respectively. DNA fragments of both fusions were excised out by digesting the plasmids with HindIII and EcoRI. After blunt ending, these fusions were cloned into HpaI site of pMV206^43^ to yield plasmids pMOHGFP1 (*pohrR*-*gfpuv*) and pMOHGFP2 (*pohr*-*gfpuv*) as shown in Figure S1. A third mycobacterial plasmid pMOHGFP3 which contains *pohr*-*gfpuv* fusion with an intact *ohrR* gene was generated as follows. First, the pMSOHR plasmid^32^ was cut with NcoI and EcoRI to release the full length *ohrR* gene with its promoter region and truncated *ohr* gene. This fragment was blunt ended and ligated to XbaI cut/blunt ended pGFPUV plasmid to yield plasmids pEOHUV3 and pEOHUV4. Plasmid (pEOHUV3) showing *ohrR*-*pohr*-*gfpuv* orientation was cut with HindIII and EcoRI, blunt ended and cloned into HpaI site of pMV206 to yield the plasmid pMOHGFP3. In addition, plasmid pMOHGFP4 was generated by cloning the *ohrR*-*pohr*-*gfpuv* fragment in the HpaI site of plasmid pMVRFP, a derivative of pMV261 that has the *rfp* gene behind *phsp60* promoter.

To determine the role of Cys residue of OhrR in sensing intracellular peroxide stress, we modified the cysteine into Arg by creating a single point mutation in the *ohrR* gene. Initially, two complementary oligonucleotides MS448M1 (5′-CTGGCCGACTTTCTGCGCTTCTCGATC TACTCG 3′) and MS448M2 (5′-CGAGTAGATCGAGAAGCGCAGAAAGTCGGCGGCCAG-3′) encompassing the sequences coding for cysteine residue of OhrR were synthesized. Using these oligonucleotides and QuikChange Site-Directed Mutagenesis Kit (Stratagene), a new plasmid (pEOHUV3m) with point mutation in *ohrR* was synthesized from the plasmid pEOHUV3. The plasmid pEOHUV3m was transformed into *E*. *coli*, amplified and the point mutation in *ohrR* verified by DNA sequencing. The *ohrR*-*pohr*-*gfpuv* region from this plasmid was released by digesting the plasmid with HindIII and EcoRI, blunt ended and ligated to pMVRFP to obtain plasmid pMOHGFP5. Further, to overexpress the mutated OhrR protein in *E*. *coli*, the coding region of mutated *ohrR* in the plasmid pEOHUV3m was amplified by the primers MS447EXF and MS447EXR, reported previously^32^, and cloned in the NdeI-BamHI site of pET16b (Novagen), resulting in plasmid p16MSOHRREX2. All plasmids used in the study are given in Table 1.

### Cell culture

Bone marrow derived macrophages (BMDMs) were isolated from the femur of C57BL/6, *PhoX* ^−/−^, *iNOS* ^−/−^ and *MPO* ^−/−^ mice (protocol #15024, approved by the Institutional Animal Care and Use Committee, Texas Tech University Health Sciences Center) and were cultured in DMEM medium with 10% fetal bovine serum (FBS; HyClone, Logan, UT) supplemented with 10 ng/ml of M-CSF at 37 °C in 5% CO_2_. RAW264.7 (TIB-71) and HeLa (CCL-2) cell lines were purchased from American Type Culture Collection (ATCC, Manassas, VA). These cells were also cultured in DMEM supplemented with 10% FBS in a 37 °C humid chamber with 5% CO_2_.

### Bacterial infection of cells

*M*. *smegmatis* strains were grown in 7H9 medium with appropriate antibiotics to log phase. They were pelleted by centrifugation, washed with sterile 1X PBS three times and resuspended in a known volume of PBS. The suspensions were passed through a 23G syringe to disperse the clumps and the colony forming units (CFUs) of the suspensions were determined. BMDM, RAW264.7 or HeLa cells (1 × 10^6^) were seeded on a glass cover slip in six well plates and grown to confluency. Bacterial strains diluted to different multiplicity of infections (MOIs) in DMEM were added to the wells containing cells and incubated at 37 °C for 4 h. Thereafter, the medium from the wells was removed and the cells washed with warm 1X PBS three times to remove the non-ingested bacteria. These wells were later filled with fresh DMEM and incubated for different time points. After the end of the experiment, the glass coverslips in the wells were washed three times with warm 1X PBS and mounted over a glass slide for microscopic examination.

### Fluorescent microscopy

To observe GFP or RFP expression in mycobacteria, strains grown in 7H9 were pelleted, washed, resuspended in PBS and induced with *t*-BHP. The bacteria were then fixed with PBS containing 4% paraformaldehyde. Approximately 15 µl of the bacterial suspension was spotted onto glass slides. This was covered with coverslips and observed under Nikon TiE Inverted Fluorescence Microscope at 60X magnification for green fluorescence using FITC filter (900 millisecond exposure), red fluorescence using Cy3 filter (400 millisecond exposure) and no fluorescence using DIC (36 milliseconds). BMDM and other cell lines grown on coverslips were infected with *M*. *smegmatis* strains, washed with PBS after incubation periods and fixed with PBS containing 4% paraformaldehyde. They were inverted onto slides and mounted for viewing under the microscope.

### Flow cytometry

BD FACSAria II was used to analyze GFP fluorescence at excitation and emission wavelengths of 395 nm and 509 nm, respectively. *M*. *smegmatis* strains fixed in 4% paraformaldehyde in PBS were directly used for flow cytometric analysis. BMDMs infected with *M*. *smegmatis* strains were scraped, treated with 4% paraformaldehyde and then analyzed. Flow data obtained was analyzed by Flow Jo software.

Overexpression of mutated OhrR. We previously reported the overexpression of recombinant *M*. *smegmatis* OhrR intact protein with His-tag (His_10_OhrR)^32^. In this study, we overexpressed the cysteine mutated OhrR* (His_10_OhrR*) using an *E*. *coli* overexpression system to compare its binding ability with *ohr*-*ohrR* promoter region. To achieve this, the plasmid p16MSOHRREX2 was transformed into *E*. *coli* strain BL21 (DE3) (Invitrogen) and induced with IPTG during log phase. After assessing the overexpression in SDS-PAGE, the cells were lysed by sonication and the His_10_OhrR* protein from the soluble fraction was purified by Ni-NTA affinity chromatography as detailed before^32, 44^.

### Electrophoretic mobility shift assay (EMSA)

As published previously, a 270 bp DNA fragment containing the *ohr*-*ohrR* intergenic region was amplified by primers MS448IGF and MS448IGR^32^. This fragment was purified by spin column and its concentration measured. Approximately, 250 ng of this DNA fragment was incubated with 500 ng of mutated His_10_OhrR* protein or unmutated His_10_OhrR protein in separate tubes for 10 min at room temperature. The protein-DNA complex was separated in 1% agarose gel with 50 mM TAE buffer. The gel was stained with ethidium bromide and photographed.

## Electronic supplementary material

Supplementary Figures
