# Supplementary material for: OhrR of Mycobacterium smegmatis senses and responds to intracellular organic hydroperoxide stress
Source: Sci Rep. 2017 Jun 20;7:3922. doi: 10.1038/s41598-017-03819-1 (PMC5478616; doi:10.1038/s41598-017-03819-1)
Supplement: Supplementary file 1 — Supplementary Figures [file 41598_2017_3819_MOESM1_ESM.pdf]

OhrR of *Mycobacterium smegmatis* senses and responds to intracellular organic hydroperoxide stress

Omar A. Garnica[1‡], Kishore Das[1‡], and Subramanian Dhandayuthapani[1*]
[1]Center of Emphasis in Infectious Diseases and Department of Biomedical Sciences, Paul L. Foster School of Medicine, Texas Tech University Health Sciences Center El Paso, Texas 79905
[‡]These authors contributed equally
*Corresponding author: s.dhandayuthapani@ttuhsc.edu, Tel: 915-215-4239

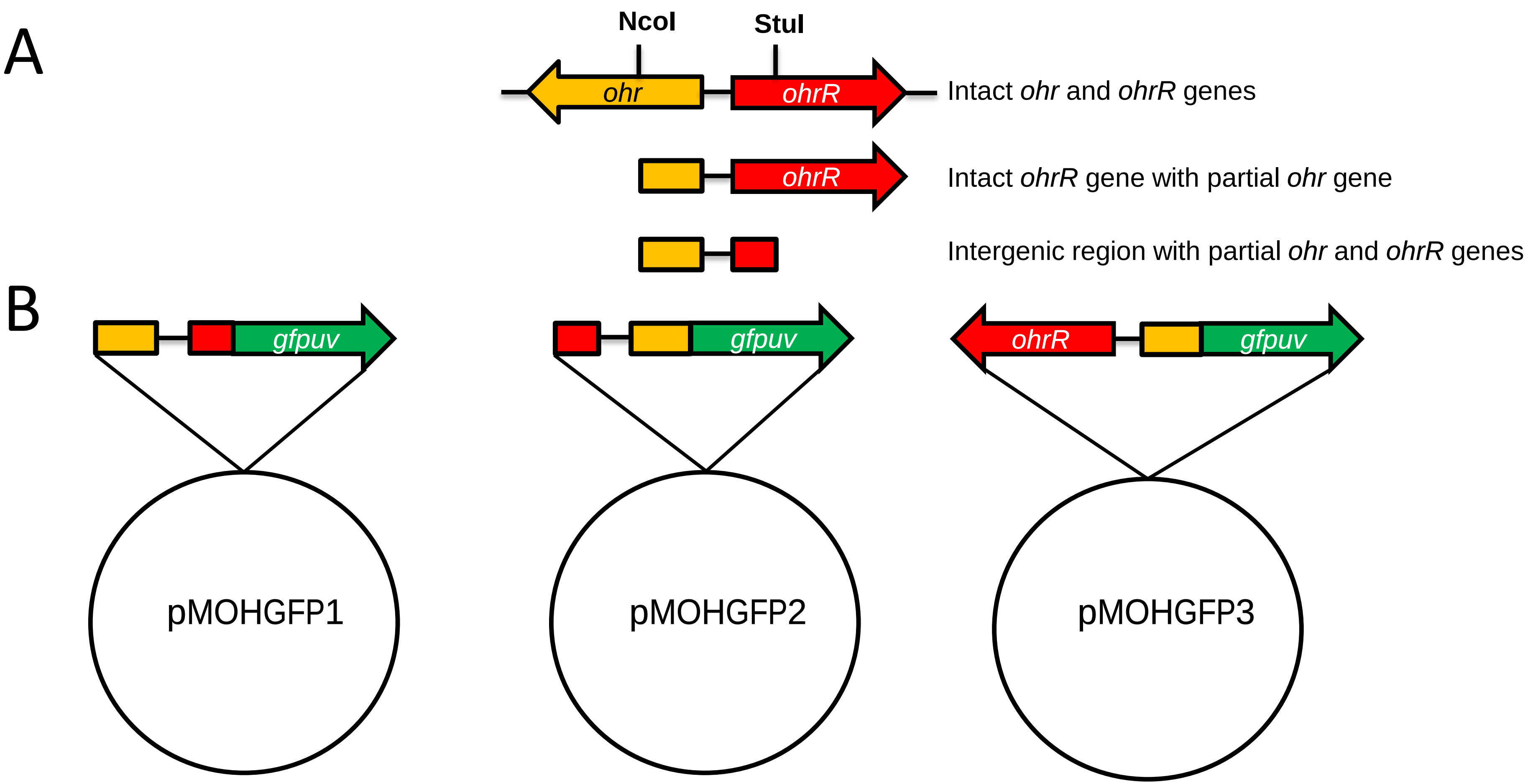

Figure S1. Schematic of *ohr-ohrR* region and GFP expressing plasmid constructs. A. Diagram showing the divergently transcribed *ohr and ohrR* genes and fragments derived from them by digesting with the restriction enzymes NcoI and StuI. **B**. Diagram showing the promoters/gene driving the expression of *gfpuv*. Plasmids pMOHGFP1, pMOHGFP2 and pMOHGFP3 bear *pohrR-gfpuv*, *pohr-gfpuv* and *ohrR-pohr-gfpuv* fusions, respectively.

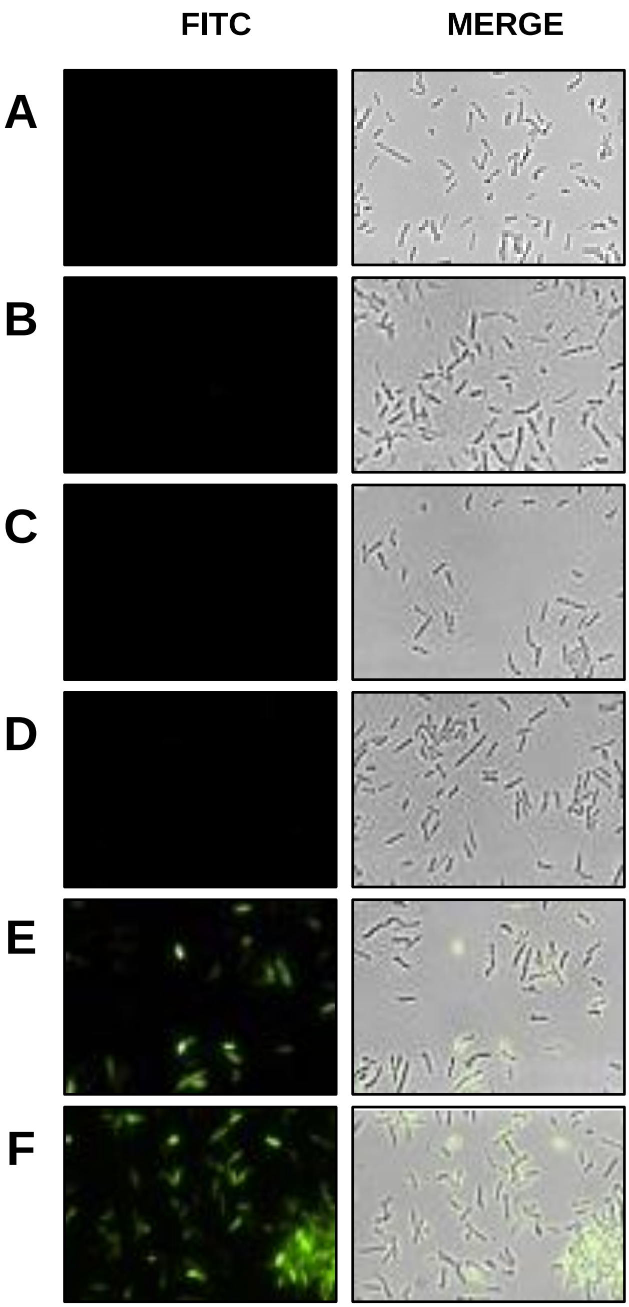

Figure S2. Induction of GFP in *M. smegmatis* strain MSOHG3 by different oxidants. MSOHG3 carrying *ohrR-pohr-gfpuv* fusion was incubated with 500 µM of different oxidants.at 37°C for 2 h. Green fluorescence in bacteria was detected using FITC filter and a 60X objective in a Nikon TiE Inverted Fluorescence Microscope as described in the methods section. **A.** un-induced control bacteria; **B-F**, bacteria induced with 500 µM hydrogen peroxide (H_2O_2), sodium hypochlorite (NaOCl), menadione, cumene hydroperoxide (CHP) and *t*-butyl hydroperoxide (*t*-BHP**),** respectively**.** FITC, images under FITC filer; MERGE, images from FITC and DIC merged.

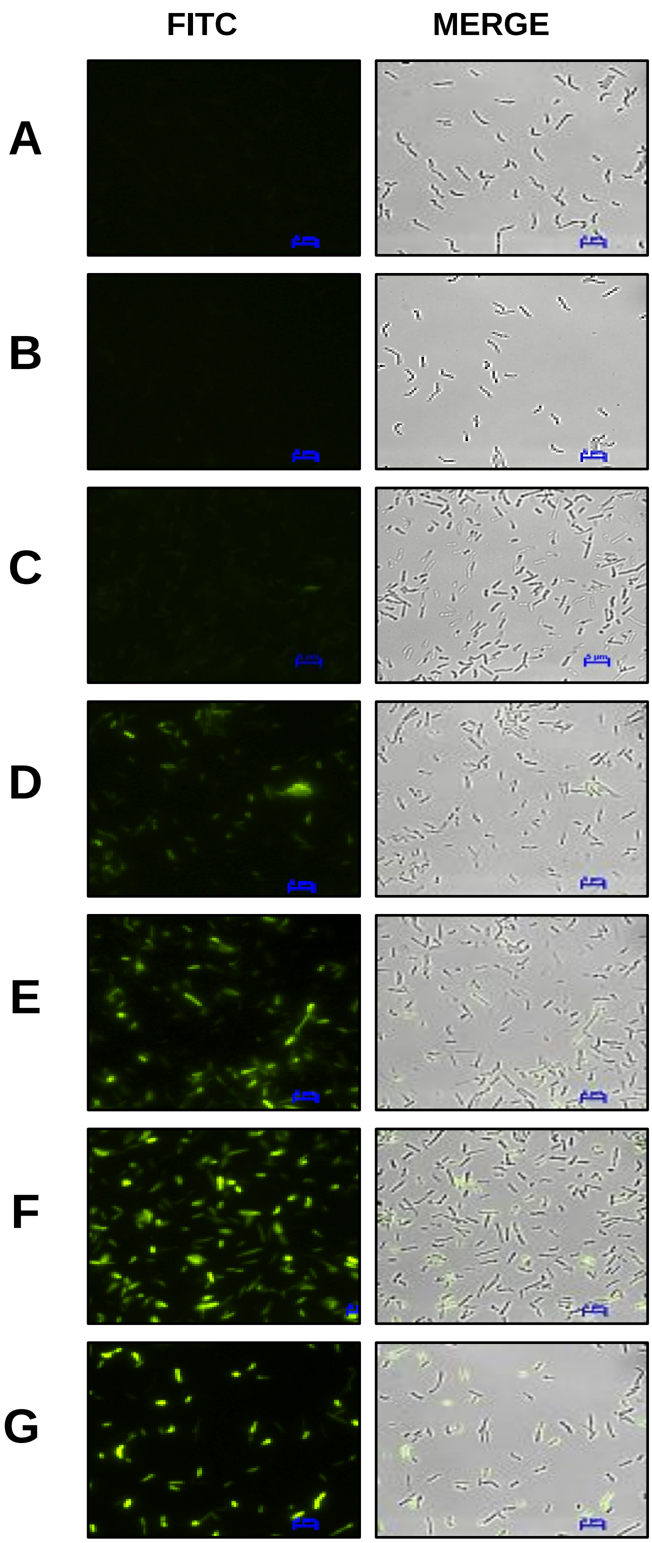

Figure S3. Induction of GFP in *M. smegmatis* strain MSOHG3 by different concentrations of *t*-BHP. MSOHG3 carrying *ohrR-pohr-gfpuv* fusion was incubated with different concentrations of *t*-BHP at 37°C for 2 h. Green fluorescence in bacteria was detected using FITC filter and a 60X objective in a Nikon TiE Inverted Fluorescence Microscope as described in the methods section. **A.** un-induced control bacteria; **B-G,** bacteria induced with 25, 50, 100, 250, 500 and 1000 µM *t*-BHP, respectively**.** FITC, images under FITC filer; MERGE, Images from FITC and DIC merged.

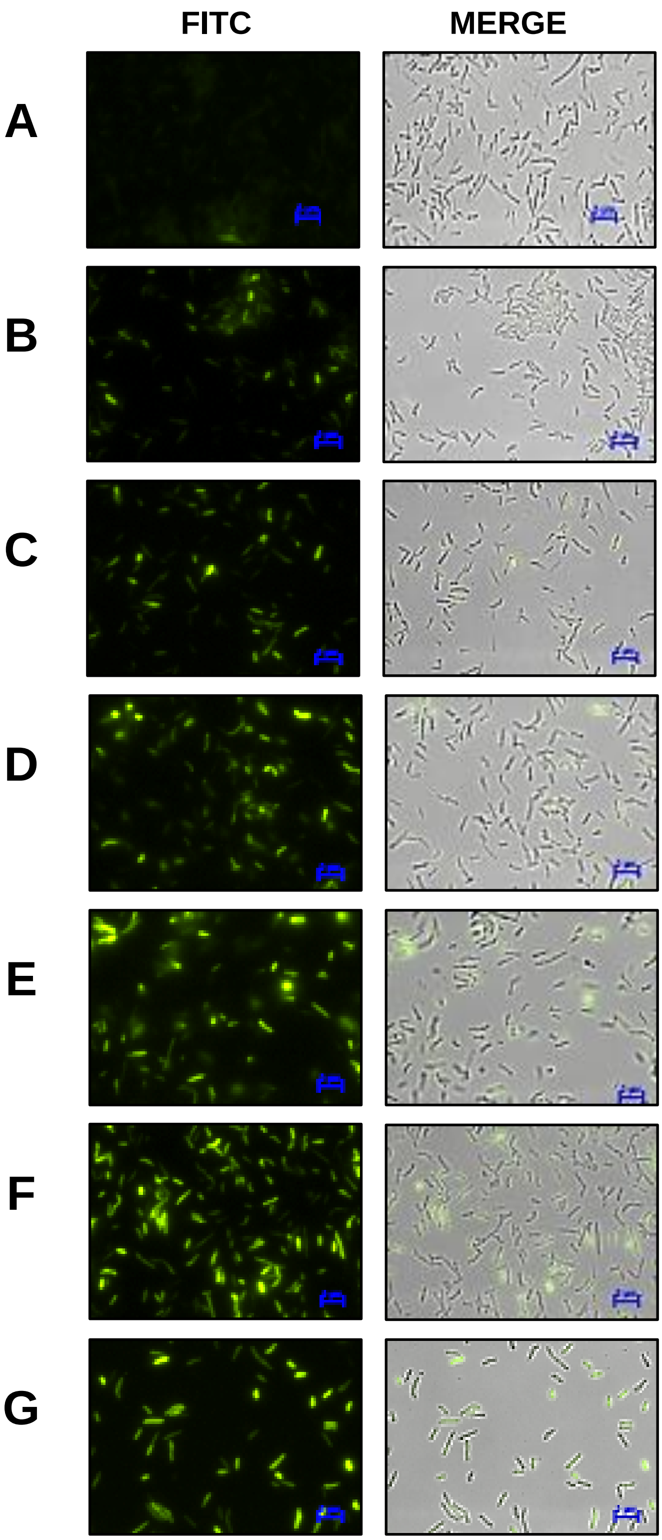

Figure S4. Induction of GFP in *M. smegmatis* strain MSOHG3 exposed to *t*-BHP for different time points. MSOHG3 carrying *ohrR-pohr-gfpuv* fusion was exposed to 100 µm of *t*-BHP for different time periods at 37°C. Green fluorescence in bacteria was detected using FITC filter with 60X objective in a Nikon TiE Inverted Fluorescence Microscope as described in the methods section. **A.** un-induced control bacteria; **B-G,** bacteria exposed to *t*-BHP for 0, 15, 30, 60, 120 and 240 minutes, respectively. FITC, images under FITC filer; MERGE, Images from FITC and DIC merged.

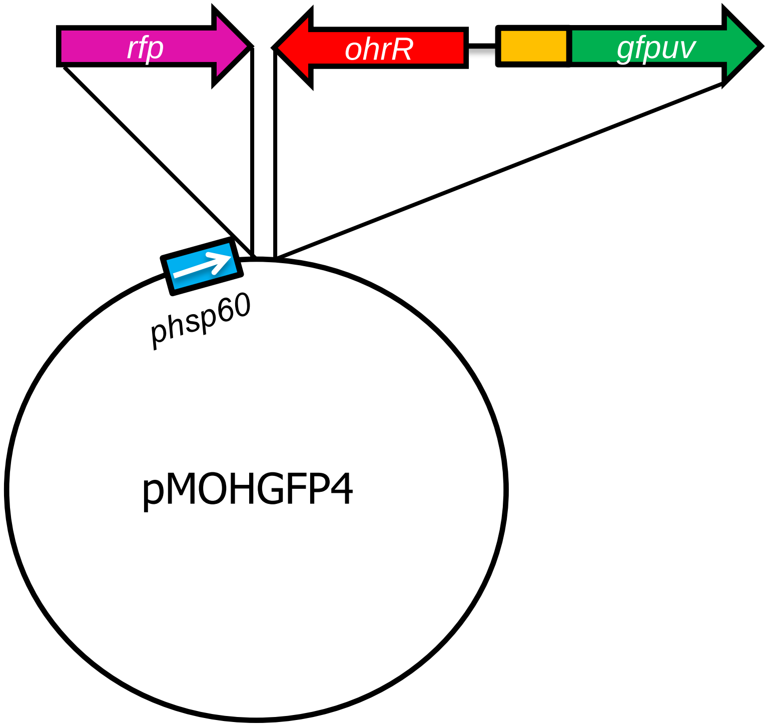

Figure S5. Schematic description of plasmid pMOHGFP4. Plasmid pMOHGFP4 was generated by cloning the *ohrR-pohr-gfpuv* fragment in the HpaI site of the plasmid pMVRFP, which is a derivative of the plasmid pMV261. In pMVRFP, the *rfp* gene encoding the red fluorescent protein is cloned behind the mycobacterial promoter *phsp60* (shown in blue). White arrow indicate the direction of *phsp60* transcription. Colored arrows indicate the direction of transcription of the genes designated on them. The rectangle shown in orange represents the coding sequences of *ohr* gene.

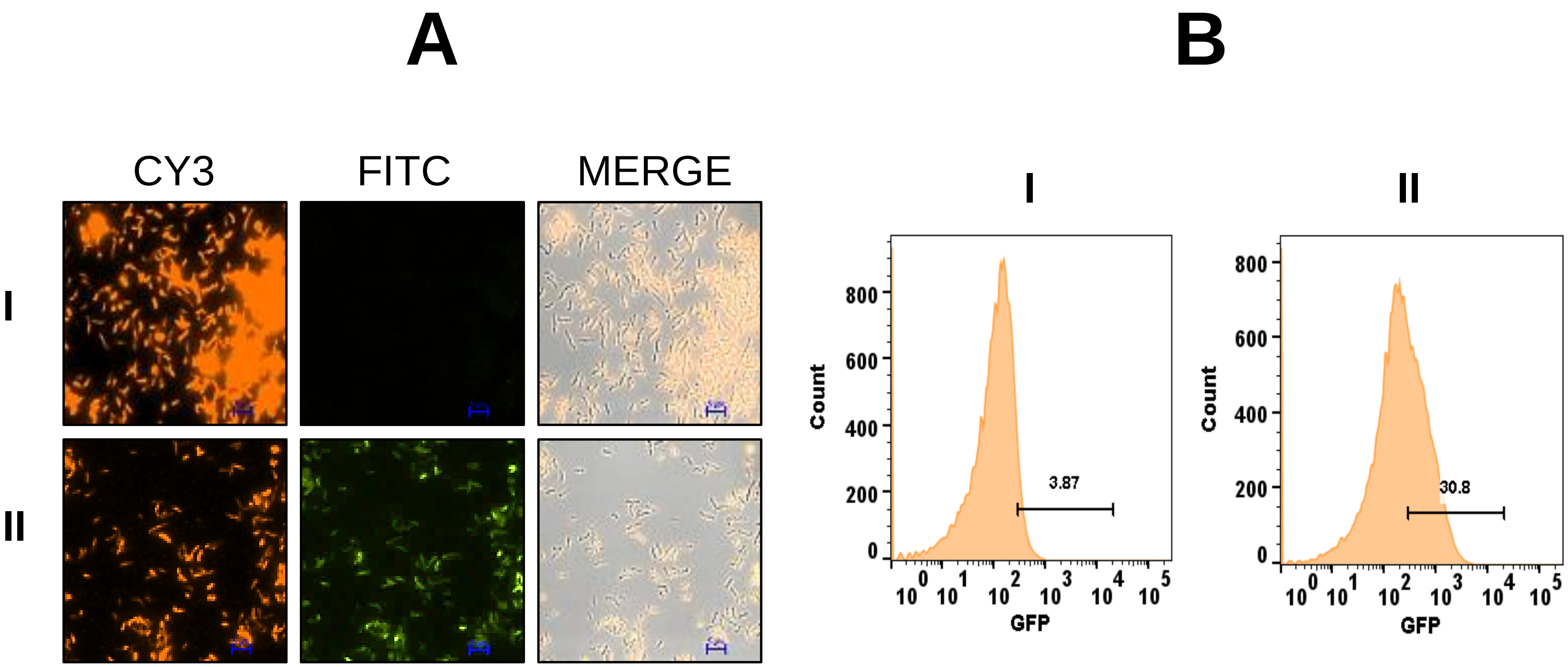

Figure S6. Induction of GFP in *M. smegmatis* strain MSOHG4 by *t*-BHP. A.. Images of Fluorescent microscopy. MSOHG4 strain, bearing *ohrR-pohr-gfpuv* and *phsp60-rfp* fusions, was induced with 500 µm of *t*-BHP and incubated at 37°C for 2 h. The bacteria were examined for GFP expression in a Nikon TiE Inverted Fluorescent Microscope using 60X objective. **I**. MSOHG4 bacteria un-induced, and **II**. MSOHG4 bacteria induced with *t*-BHP. Cy3 and FITC indicate images obtained using these filters. MERGE indicates the merged images of CY3, FITC and DIC. **B**. **Flow cytometry**. MSOHG4 was exposed to 500 µm of *t*-BHP for 2 h at 37°C. Flow analysis was performed in BD FACSAriaII using excitation and emission wavelengths of 395nm and 509nm, respectively. **I.** un-induced MSOHG4 bacteria, II. MSOHG4; bacteria exposed to *t*-BHP.

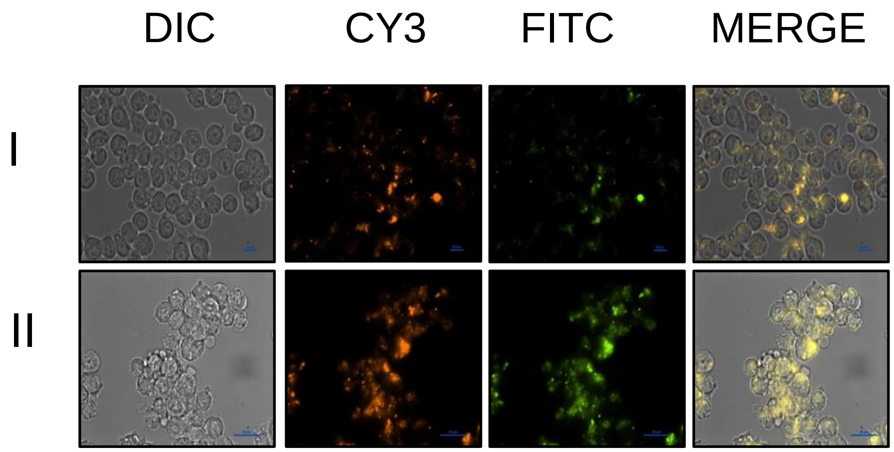

Figure S7. Induction of GFP in *M. smegmatis* MSOHG4 strain by intracellular organic hydroperoxides of RAW264.7 macrophages. RAW264.7 cells grown on glass coverslips were infected with *M. smegmatis* MSOHG4 bearing *ohrR-pohr-gfpuv* and *phsp60-rfp* fusions for 4 h or 24 h at 37°C. After washing, the coverslips were examined under Nikon TiE Inverted Fluorescence Microscope using 60X objective. DIC, Cy3 and FITC indicate images obtained using these filters. MERGE indicates the merged images of DIC, CY3 and FITC. I and II, images of RAW264.7 infected with MSOHG4 after 4 h and 24 h of infection, respectively.
